# Does attentional selectivity in global/local processing improve discretely or gradually?

**DOI:** 10.3389/fpsyg.2014.00061

**Published:** 2014-02-03

**Authors:** Ronald Hübner

**Affiliations:** Fachbereich Psychologie, Universität KonstanzKonstanz, Germany

**Keywords:** global/local processing, binding, attentional selectivity, sequential sampling, early and late selection

## Abstract

Some results suggest that attentional selection in global/local processing occurs at two stages: an early stage, where global and local information of a hierarchical stimulus is filtered or weighted according to the current goal, and a late stage, where the contents of the stimulus are bound to their respective level. Because it is assumed that binding improves attentional selectivity, accuracy should increase with response time. To see whether this prediction holds, a global/local experiment was conducted with hierarchical letters as stimuli, and where selection difficulty was varied by blocking vs. randomizing the target levels. The results show that accuracy indeed increased with response time, although to a lesser extent under randomized levels. Because an increasing accuracy is also compatible with a gradually improving selectivity, corresponding sequential sampling models were fit to the distributional data. The results show that a discretely improving attentional selectivity accounts better for the data. Moreover, the parameters of the corresponding model indicate that randomizing the target level impaired the efficiency of early selection as well as that of content-to-level binding.

## Introduction

**Figure 1:**
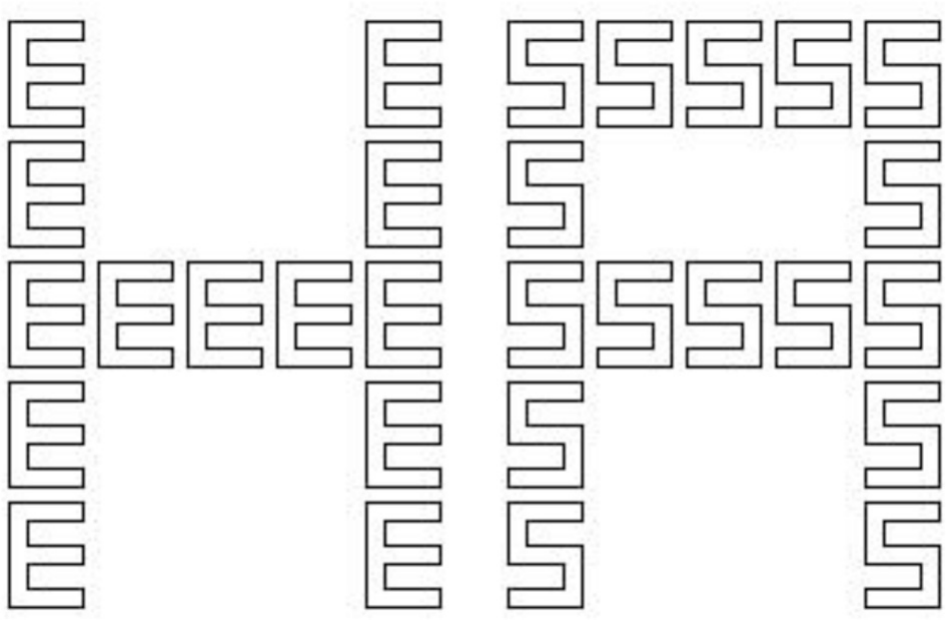
**Two examples of the hierarchical letters presented in the experiment**. Both stimuli are incongruent. In the experiment the stimuli were presented in white on a black background.

Many objects in our environment not only consist of multiple parts, but also have a hierarchical structure. For instance, human bodies are composed, beside other parts, of arms, which, in turn, are composed of hands, fingers, etc. An important question therefore is how such objects are perceived and represented. Does the visual system first process the parts and then use the results to construct a mental representation of the whole object (e.g., Wundt, 1874)? Or is the global shape of the object perceived first and then parsed into its components, as assumed by Gestalt psychologists (e.g., Wertheimer, 1922, 1923)? Great progress concerning this question has been made after Navon (1977) introduced hierarchical letters as stimuli (see Figure 1). By presenting these stimuli and requiring his participants to either identify the letter at the global or the local level, he found that responses to global letters were faster and more reliable than those to local ones. Moreover, the interference from global to local was larger than vice versa. These results led Navon to propose his *perceptual* global precedence hypothesis, which states that global features are processed before local ones by early perceptual mechanisms. Later studies, however, found that the level that is preferentially processed depends, besides the participants' expertise (Beaucousin et al., 2011), on various stimulus factors such as global/local size ratio, local density, retinal position, spatial uncertainty, etc. (for an overview see Kimchi, 1992).

Furthermore, it has been shown that *selective attention* also plays an important role for the relative strength of the stimulus levels (e.g., Miller, 1981; Boer and Keuss, 1982). For instance, Miller (1981) found that information from both levels is available at the same time and processed in parallel. He therefore concluded that a global advantage results from the fact that global information is usually more salient and attention grabbing than local information. For goal-directed behavior, however, response selection cannot rely exclusively on stimulus-driven processes. If this were the case, then the salient level would always determine the response. In case of incongruent stimuli, i.e., stimuli whose information at one level activates a response opposite to that activated by the information at the other level, this would always lead to an error if the goal-relevant (target) level is less salient than the irrelevant level. Therefore, selective attention is usually required to favor information processing at the target level.

A possible mechanism for attentional selection is perceptual filtering of relevant physical stimulus attributes (Desimone and Duncan, 1995). Because local and global units differ in absolute physical size, a spatial filter might be applied for selection (Lamb and Robertson, 1988), operating analogous to a zoom lens (Eriksen and St. James, 1986; Müller and Hübner, 2002). By adjusting the diameter of the zoom lens, processing can be biased in favor of the one or the other level. Moreover, global and local information also differ in their spatial-frequency content. Accordingly, it has been hypothesized that spatial-frequency filtering is used for selecting information from a certain level (Ivry and Robertson, 1998). Such an idea is not unreasonable, because it has been shown that attention can be focused on individual spatial-frequency channels (e.g., Hübner, 1996a,b). Thus, different stimulus features seem to be appropriate for attentional filtering.

From what we have considered so far, a simple model that might account for many of the reported results seems to be straightforward. Information extracted from either stimulus level feeds simultaneously into a decision process and the relative contribution of the individual levels to the decision depends on stimulus-dependent as well as intentional factors. Goal-directed behavior is accomplished by biasing, i.e., by adjusting early attentional filters in such a way that information at the target level is processed preferentially and, consequently, determines the response.

However, such a biasing account alone is not sufficient to explain all available data. The reason is that early perceptual filtering is not effective under all circumstances. Filtering might be sufficient if stimulus position and target level remain constant across trials, because in this case it is easy to adjust the zoom lens or a spatial-frequency filter in such a way that information at the target level dominates. In other situations, however, filtering is not sufficient for a reliable performance. One reason is that attention is not fully under voluntary control and also depends, among others, on its previous state. For instance, if the target level varies randomly across trials, so that individuals have frequently to switch between levels, then attention can be adjusted only suboptimally, which is reflected by a reduced performance and an increased interference between the levels (Hübner, 1997, 2000).

Considering these selection difficulties and other results, Hübner and Volberg (2005) concluded that a simple filter model, as outlined above, is insufficient as a general account of global/local processing, and that, to guarantee a reliable goal-directed performance, an additional mechanism is required. In their *content-to-level binding* (CLB) theory they proposed such a mechanism. They assumed that, at the beginning of stimulus processing, and possibly modulated by filtering or biasing, the contents of the different levels in a hierarchical object are identified and represented independently of their respective level. Consequently, to obtain a complete object representation that can be used, for instance, to resolve response conflicts, the contents have subsequently to be actively linked to their respective level. This view also agrees with neuroscientific results showing that the processing of information at early stages is affected by the output of processes at later stages (e.g., Hon et al., 2009).

The idea of content-to-level binding is specifically supported by results from experiments in which participants had to indicate the identity of the letter at a pre-specified target level of a hierarchical letter that was masked shortly after its presentation (Hübner and Volberg, 2005). If the CLB theory is right, then masking should disturb the binding process, which, in turn, should produce binding errors between levels and identities (Treisman and Gelade, 1980). Consequently, participants should frequently report the letter at the non-target level, which is indeed the case (e.g., Hübner and Volberg, 2005; Flevaris et al., 2010; Hübner and Kruse, 2011).

This short overview shows that global and local information of a hierarchical object are processed in parallel, and that the relative strength of processing in a given pathway depends on stimulus features as well as on attentional adjustments. Moreover, if goal-directed response selection is difficult, e.g., because attentional biasing or filtering is ineffective, a further selection process is needed. According to CLB theory, this process binds the different contents to their respective level. Thus, it seems that there are two stages of stimulus selection: an early stage and a late stage. Interestingly, two selection stages have also been proposed for the flanker task (Gratton et al., 1992; Hübner et al., 2010) to account for the phenomenon that accuracy for incongruent stimuli usually increases during response selection. As far as I know, global/local processing has not yet been analyzed in this respect. Therefore, one aim of the present study was to investigate whether accuracy increases in this task as well. As we will see, this is indeed the case, which indicates that stimulus selectivity (i.e., the ability to restrict perceptual processing to the task-relevant item)also improves during global/local processing.

A further aim of the present study was then to investigate *how* stimulus selectivity improves. As already mentioned, according to the CLB theory, selectivity increases abruptly. After stimulus onset attentional selectivity obtained by filtering or biasing is relatively low, but then advances due to the output of a late stage, where stimulus contents are bound to their levels. However, one might question that selectivity improves in a single step. An alternative idea is that selectivity increases gradually. For instance, one could assume that early mechanisms, i.e., perceptual filtering, improve steadily during stimulus processing. The questions of whether a discrete or a gradual increase of selectivity accounts better for the performance in global/local tasks should be answered by applying corresponding sequential sampling models. The discrete increase was represented by the dual-stage two-phase (DSTP) model (Hübner et al., 2010), whereas the “shrinking-spotlight” (SSP) model (White et al., 2011) exemplified the gradual increase.

The DSTP model (Hübner et al., 2010), which is described in detail in the Modeling section, includes an early and a late stage of attentional stimulus selection (for a graphical illustration see Figure 5). At the early stage, information is weighted according to its relevance. For instance, if the target level is local, then the attentional weight for information transmitted through the “local channel” is increased relative to that for the “global channel.” The weighted information determines the initial rate of evidence accumulation. Selectivity is then further improved by an additional (late) stimulus-selection process that proceeds by content-to-level binding. If successful, the rate of evidence is increased, as in our example in Figure 5.

The SSP model (White et al., 2011) has specifically been developed to account for flanker-task data (see also Hübner and Töbel, 2012). However, the metaphor of a gradually shrinking spotlight is similar to the idea of a zoom lens and, therefore, can easily be adapted to the global/local task. For instance, if the target level is local, then one could assume that the attentional “spotlight” initially encompasses the global shape of the stimulus and then shrinks until it encloses only a single local item. Due to the shrinking, selectivity improves continuously. For changing the focus of attention in the opposite direction, one would have to assume that the “spotlight” can also expand. In any case, more abstractly, one can assume that selectivity generally improves gradually in the direction of the target level (for a more detailed description of the SSP model see below).

## Experiment

To get a deeper insight into the dynamics of information representation and selection in global/local tasks and the involved processes an experiment was designed, in which the participants had to categorize the letter at a pre-cued target level, and where task difficulty was varied by blocking vs. randomizing the target level. It was expected that information selection is generally less efficient under randomized levels (Hübner, 1997; Hübner et al., 2001), which should be observable by an increased interference (congruency effects) between the levels. Moreover, accuracy should improve more slowly with RT when the target level varies across trials. Whether this is the case should be examined by considering so-called *conditional accuracy functions* (CAFs).

To examine how selectivity changes during processing, the DSTP model and the SSP model should be fit to the distributional data. If stimulus selectivity improves gradually, then the SSP model should generally fit the data better than the DSTP model. Given this is not the case, then, with respect to the DSTP model it was expected that late selection (content-to-level binding) plays a greater role when target level varies across trials, which should be reflected by differences between the corresponding model parameters.

### Method

#### Participants

17(mostly psychology) students from the Universität Konstanz, Germany, participated in the experiment. All had normal or corrected-to-normal vision, were right-handed by self-report, and were paid 8 € for their participation. One student was excluded from analyses because of his high error rate (>25%). The remaining 16 participants (13 females) had a mean age of 25 years. The experiment was performed in accordance with the ethical standards laid down in the 1964 Declaration of Helsinki and its later amendments. In agreement with the ethics and safety guidelines at the Universität Konstanz, we obtained a verbal informed consent statement from all individuals prior to their participation in the study. Potential participants were informed of their right to abstain from participation in the study or to withdraw consent to participate at any time without reprisal.

#### Apparatus and stimuli

Stimuli were presented on a 18″ color-monitor with a resolution of 1280 × 1024 pixels and a refresh rate of 60 Hz. Stimulus presentation as well as response registration was controlled by the same personal computer (PC).

For constructing hierarchical letters (for examples see Figure 1), four different letters (A, S, H, E) were used and divided into two response categories (E, S and A, H). By combining all letters, 16 hierarchical letters were created, where global letters were constructed from identical local letters in a 5 × 5 grid. A stimulus was congruent if the letters at both levels belonged to the same response category; otherwise it was incongruent. The size of the global letters was 4.48° of visual angle horizontally and 5.72° vertically. The respective size of the local letters was 0.72° × 1.08°. The local letters were constructed by outlines. Stimuli were presented in white on a black background.

#### Procedure

**Figure 2:**
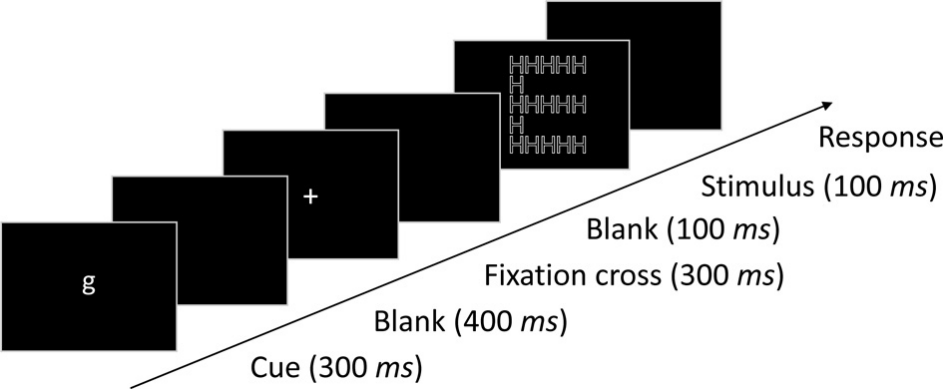
**The procedure applied in the experiment**. In this example the cue (g) indicates global as target level.

Participants were seated at a viewing distance of approximately 60 cm in front of the screen. A trial started with the presentation of a cue (the letter “*l*” or “*g*” to indicate a local or global target level, respectively) at the center of the screen for 300 ms, followed by a blank screen for 400 ms. After the presentation of a fixation cross for 300 ms and a subsequent blank screen of 100 ms the stimulus was presented for 100 ms at the center of the screen. The next trial started 1000 ms after the response. An illustration of the procedure is shown in Figure 2. The task of the participants was to identify the response category of the letter at the cued target level by pressing one of two response buttons of a computer mouse. After some training (1 block of 32 trials), 28 blocks of 64 trials for each participant were run in a single 1 h session. This resulted in 224 trials per condition. Blocks with constant-level and randomized-level alternated. The block type at the beginning was balanced across participants.

### Results and discussion

Responses faster than 100 ms or slower than 1200 ms were excluded from data analysis (<0.15% of all data).

The mean latencies of correct responses were subjected to a three-factor ANOVA for repeated measurements on the factors *block type* (constant level, or randomized level), *target level* (global, or local), and *congruency* (congruent, or incongruent). The analysis revealed significant main effects of *block type*, *F*_(1, 15)_ = 62.8, *p* < 0.001, η^2^_*p*_ = 0.807, and *congruency*, *F*_(1, 15)_ = 98.5, *p* < 0.001, η^2^_*p*_ = 0.868. Responses were faster for a constant target level, compared to a randomized one (511 vs. 553 ms), and for congruent compared to incongruent stimuli (521 vs. 543 ms). However, there was also a significant interaction between *block type* and *congruency*, *F*_(1, 15)_ = 14.5, *p* < 0.01, η^2^_*p*_ = 0.492, indicating that the congruency effect was smaller under constant levels, compared to randomized ones (Δ15 vs. Δ29 ms). Moreover, there was an interaction between *target level* and *congruency*, *F*_(1, 15)_ = 10.8, *p* < 0.01, η^2^_*p*_ = 0.420. The congruency effect was smaller for the local compared to the global target level (Δ 18 vs. Δ 28 ms).

Mean error rate was 8.81%. Subjecting the error rates to an ANOVA of the same type as for the mean RTs also revealed significant main effects of *block type*, *F*_(1, 15)_ = 10.6, *p* < 0.01, η^2^_*p*_ = 0.415, and *congruency*, *F*_(1, 15)_ = 93.7, *p* < 0.001, η^2^_*p*_ = 0.862. The error rate was smaller when the target level was constant, compared to when it was randomized (7.87 vs. 9.785%), and smaller for congruent than for incongruent stimuli (6.15 vs. 11.5%). However, *congruency* interacted significantly with *block type*, *F*_(1, 15)_ = 5.28, *p* < 0.05, η^2^_*p*_ = 0.260, indicating that the congruency effect was smaller for constant target levels than for randomized ones (Δ 4.02 vs. Δ 6.63%). Finally, the three-way interaction between all factors was significant, *F*_(1, 15)_ = 6.66, *p* < 0.05, η^2^_*p*_ = 0.308. It indicates that the congruency effect was larger for the global target level than for the local one, but only under randomized levels(constant: global Δ 3.95% vs. local Δ 4.09%; randomized: global Δ 8.56% vs. local Δ 4.70%).

Obviously, there was no global advantage in the experiment, which is in line with other studies using similar stimulus conditions (e.g., Volberg et al., 2009). One crucial condition was that the stimuli were presented at the center of the screen. Moreover, the applied outline letters possess a relatively high proportion of high spatial frequencies (Hübner and Kruse, 2011). Together, these features favored local processing, so that there was even a small but reliable local advantage in RT with respect to congruency. In the error rates the local advantage in congruency occurred only under randomized levels. Furthermore, as expected, randomizing the target levels generally reduced performance in RT and the error rates.

#### Distributional data

For inspection and modeling, cumulative distribution functions (CDFs) were constructed for the latencies of correct responses. Error data were represented by conditional accuracy functions (CAFs), because they directly show how accuracy (selectivity)varies with RT, and because, in contrast to CDFs for error RTs, CAFs can also be computed for participants that produce few or no errors in some conditions, e.g., for congruent stimuli.

**Figure 3:**
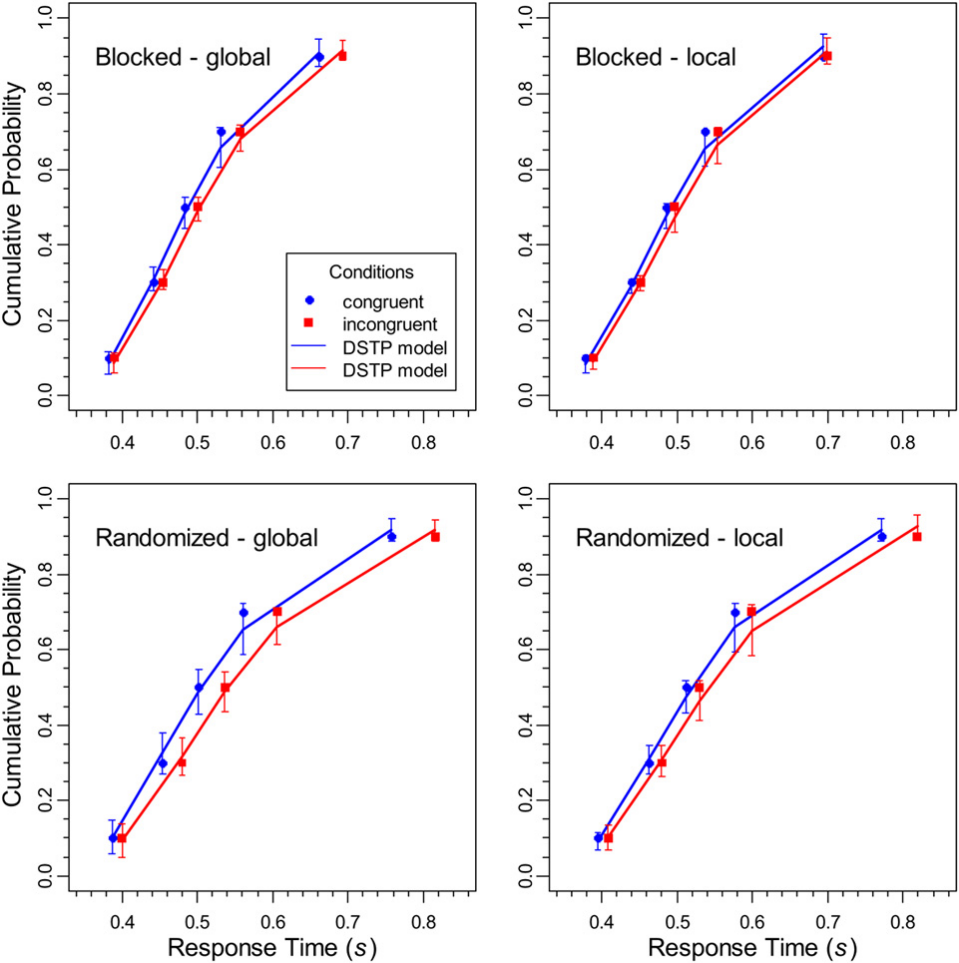
**The data points represent the cumulative distribution functions for the RTs of correct responses for the different conditions in the experiment, whereas the solid lines show the respective performance of the DSTP model**. The error bars represent the 95%-confidence intervals of the theoretical function, estimated from the results of the jackknife procedure.

CDFs were constructed for each block type (constant target level, randomized target level), target level (global, local), and congruency condition (congruent, incongruent) by quantile-averaging (0.1, 0.3, 0.5, 0.7, and 0.9) the corresponding data (Ratcliff, 1979). By this procedure, the data for each participant, condition, task, and response type were sorted into 6 bins comprising 10, 20, 20, 20, 20, and 10% of the data, respectively. The resulting CDFs are shown in Figure 3. As can be seen, for most conditions the functions diverge, which is similar to the results obtained with the flanker task (Hübner et al., 2010) and shows that the congruency effect in the latencies increased with RT. Furthermore, it is obvious that randomizing the targets level had its main effect on slower responses. The fastest correct responses were hardly affected.

**Figure 4:**
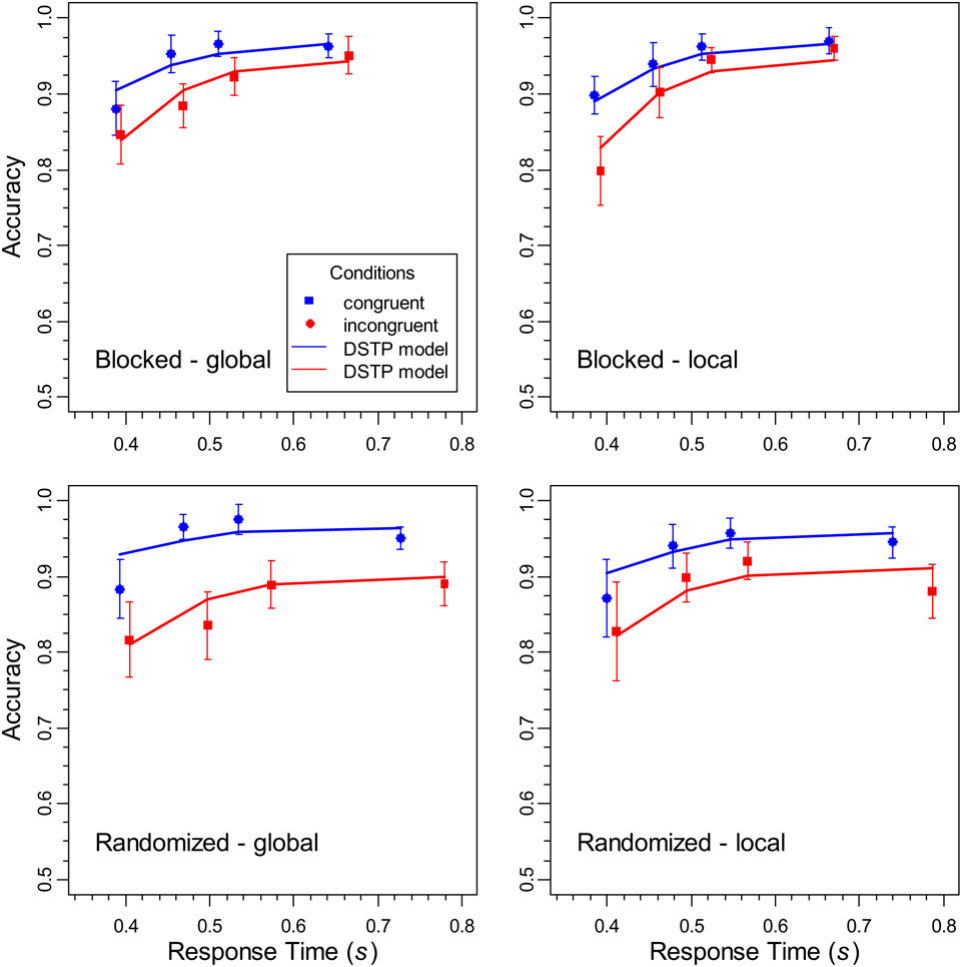
**The data points represent the conditional accuracy functions for the different conditions in the experiment, and the error bars show the 95%-confidence intervals**. The solid lines represent the corresponding performance of the DSTP model.

CAFs were constructed by sorting the data of each participant, condition, and task into four 25% bins, and by calculating the mean RT and proportion of correct responses for each bin. The obtained values were then averaged across participants (e.g., Ridderinkhof, 2002). The CAFs are shown in Figure 4. It can be seen that accuracy was lowest for the fastest responses to incongruent stimuli. However, it increased with RT and almost approached the same high level as the accuracy for congruent stimuli, at least under constant target levels. In contrast, when the target level was randomized, some congruency effect remained also for slow responses. Together, these data demonstrate that selectivity also improves with RT in the global/local task, although the improvement is limited under randomized levels.

### Modeling and discussion

The DSTP model and the SSP model were fit to the distributional data for assessing model performance and for estimating the respective parameter values for the different conditions. To understand the specific roles of the different model parameters, a more detailed description of the respective model is provided before the corresponding fit results are reported.

#### The DSTP model

**Figure 5:**
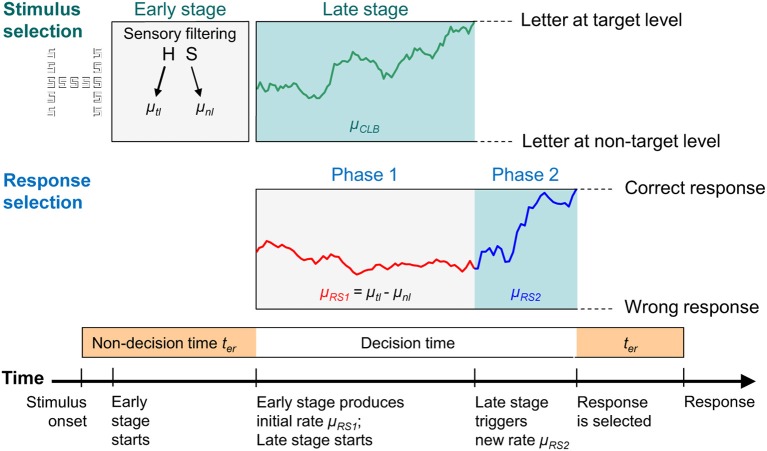
**A graphical illustration of the dual-stage two-phase (DSTP) model**. An early stage of stimulus selection (i.e., sensory filtering/weighting) provides component rates according to the information at the target level (μ_*tl*_) and at the non-target level (μ_*nl*_), which sum up to the drift rate μ_*RS1*_ for Phase 1 of response selection. Because the stimulus is incongruent in this example, μ_*nl*_ is negative. In parallel with response selection in Phase 1, a late stimulus selection process (*CLB*) runs with rate μ_*CLB*_ until it reaches one of the two boundaries. Here, the upper boundary was hit, which leads to the binding of the target letter to the target level. After binding, response selection enters Phase 2, which is characterized by a new (higher) drift rate μ_*RS2*_. The decision is completed as soon as the response selection process hits one of the two response boundaries reflecting the choice alternatives. The duration for the non-decisional processes (sensory filtering, motor execution, etc.) is captured by the parameter *t*_*er*_.

The core of the DSTP model is response selection, which is divided into a first and a second phase (Phase 1 and Phase 2), each represented by a diffusion process (cf. Ratcliff, 1978; Ratcliff and Rouder, 1998) *RS1* and *RS2*, respectively (for a graphical illustration see Figure 5). Basically, a diffusion process is characterized by a drift rate parameter, reflecting the evidence available for responses A and B, and by two corresponding threshold parameters *A* and –*B*. Noisy samples of evidence are accumulated in time, beginning at state *X*_0_ until threshold *A* or –*B* is reached, which then triggers the corresponding response. It is assumed that *X*_0_ = 0, and that A and B represent the correct and wrong button press, respectively.

In the first phase of response selection, evidence (for driving *RS1*) is provided by an early stage of information selection, where global and local stimulus features (e.g., spatial areas or spatial frequencies) or corresponding channels are biased or weighted in favor of the target level. The product of stimulus information and the respective attentional weights are represented by component rates μ_*tl*_ and μ_*nl*_. The parameter μ_*tl*_ represents the rate of evidence provided by the target level in favor of the correct response, whereas μ_*nl*_ stands for the evidence contributed by the information at the non-target level for that response. Both rates sum up to the total rate μ_*RS1*_ for process *RS1*, i.e., μ_*RS1*_ = μ_*tl*_ + μ_*nl*_. The sign of μ_*nl*_ is positive if the stimulus is congruent, but negative if it is incongruent. Thus, the overall rate for *RS1* is reduced for incongruent stimuli, compared to congruent ones, and can even be negative.

If response selection would simply proceed by process *RS1* alone, then accuracy would remain at a relatively low level. However, as we have seen, at least for constant target levels, accuracy improved substantially with RT (see Figure 4). To model this improvement, a further and more sophisticated late-selection process (here, content-to-level binding, CLB) is running in parallel with *RS1*. The dynamics of late selection is also implemented as a diffusion process with rate μ_*CLB*_. It is assumed that it binds either C, the content of the target level, or D, the contend of the non-target level to the target level, depending on whether it hits threshold *C* or –*D*, respectively. Thus, selecting D by late selection represents a binding error, i.e., the event that the content of the non-target level was erroneously linked to target level.

If the late-selection process finishes before a response is selected by *RS1*, then, from that point onwards, response selection enters Phase 2 and continues by process *RS2* (see Figure 5). The rate of *RS2* depends on the binding result. If the information at the target level was linked to the target level, then the rate is μ_*RS2*_. Due to the assumed high quality output of the binding process, the rate for *RS2* is usually higher than that for *RS1* in Phase 1. Such a situation is shown in the example in Figure 5. In case the information at the non-target level was erroneously linked to the target level, the rate depends on the stimulus type. If the stimulus is congruent, then the rate is also μ_*RS2*_. However, if the stimulus is incongruent, then the rate μ_*RS2*_ is negative, which produces an error with high probability.

For the specific model version applied in this study, the number of free parameters was reduced by assuming symmetric thresholds for response and information selection. Consequently, the model has 7 parameters: Thresholds *A* = *B* for response selection in Phase 1 and Phase 2; the component rates for the target and non-target level, μ_*tl*_ and μ_*nl*_; the rate μ_*RS2*_ for *RS2*; the rate, μ_*CLB*_, and thresholds *C* = *D* for the binding process; and finally, a non-decisional parameter, *t*_*er*_, representing the time consumed by non-decisional processes (filtering and motor processes).

#### Fit procedure

A computer-simulation version of the DSTP model was fit to the CDFs for correct responses and to the proportion of errors in the bins of the CAF for a given condition. In all, for each of the four main conditions (target-level × block type)there were 6 bins for correct responses to congruent stimuli, 4 bin for errors in the congruent condition, 6 bins for correct responses to incongruent stimuli, and 4 bins for errors in the incongruent conditions.

The fit procedure was the same as in Hübner et al. (2010). Specifically, the PRAXIS algorithm (Brent, 1973; Gegenfurtner, 1992) was applied to find parameter values that minimized the *G*^2^ (Wilks likelihood ratio chi-square) squared statistics (cf. Ratcliff and Smith, 2004):

$$G^{2}=2\sum_{i=1}^{J}Np_{i}\ln\left(\frac{p_{i}}{\pi_{i}}\right),$$

In this equation, *J* is the number of bins, *p_i_* is the proportion of observations in the *i*th bin, and π_i_ is the proportion in this bin predicted by the considered model. *N* is the number of all observations. For computing the degrees of freedom (*df*) of the goodness-of-fit statistics, let *J* be the number of bins for each main condition, respectively, and *M* the number of model parameters. We then have *df* = 2(*J* − 1) − *M*. For the DSTP model with 7 parameters we have *df* = 2(10 − 1) − 7 = 11.

For *N* the average number of valid trials per person in the corresponding fit condition was used. This was uncritical in the present case, because *G*^2^ was not appropriate for significant testing, and merely served as goodness-of-fit measure (cf. Ratcliff and Smith, 2004). Starting from different sets of parameter values to avoid local minima, each fit was continued until *G*^2^ was minimized. For each of the required several hundred cycles, 8 × 10^5^ trials were simulated.

To be able to test parameter differences between the conditions, some measure of parameter variability was needed. One way would have been to fit the model to individual data. However, the data of a given participant are rather noisy, which makes model fitting difficult. Therefore, a jackknife procedure (e.g., Gray and Schucany, 1972) was applied. By this procedure a jackknife subsample P_*i*_ of parameter values was computed for each participant *i* (*i* = 1·s *N*) by temporarily omitting participant *i* and by fitting the model to the averaged data computed from the remaining *N* − 1 participants. That is, the set of parameter values *P*_*i*_ are the parameters obtained by fitting the model to the average data including all participants except participant *i*. This procedure was repeated for each subject and the mean values across these subsamples were then taken as estimates of the parameters. The subsamples for each parameter were then entered into an ANOVA for repeated measurements on the factors *block type* (constant level, or randomized level), and *target level* (global, or local). The resulting artificially large *F*-values had then to be corrected according to the formula: *F*_*c*_ = *F*/(*N* − 1)^2^, where *Fc* is the corrected *F* value (Ulrich and Miller, 2001).

#### Fit results for the DSTP model

The theoretical CDFs for correct responses and the CAFs are shown as line graphs in Figures 3 and 4, respectively. The theoretical data were computed by simulating the model performance with the means of the estimated parameter values, which are shown, together with the goodness-of-fit measures in Table 1. As can be seen, the DSTP model fit the data rather well.

**Table 1:** **Parameter estimates obtained by fitting the DSTP model to the distributional data of the four conditions in the experiment**.

		**Parameters**								
**Target level**	**μ_tl_**	**μ_nl_**	***A*/*B***	**μ_CLB_**	***C/D***	**μ_RS2_**	**t_er_**	***G*^2^**	***df***	***BIC***
**CONST.**										
Global	0.1501	0.0261	0.0597	0.3160	0.0904	1.204	0.3018	7.90	11	50.6
Local	0.1301	0.0225	0.0619	0.3210	0.0865	1.020	0.2917	10.4	11	53.1
**RAND.**										
Global	0.1431	0.0440	0.0648	0.1986	0.0884	0.938	0.2920	14.9	11	57.5
Local	0.1300	0.0297	0.0651	0.2190	0.0861	0.971	0.2977	14.1	11	56.7
	L^***^	L^*^; B^**^		B^***^; L^***^; B×L^***^		B^***^; L^*^; B×L^*^				

μ_tl_, component rate for the target level; μ_nl_, component rate for the non-target level; A/B, response thresholds; μ_CLB_, rate for level selection; μ_RS2_, rate for response selection in Phase 2; t_er_, mean non-decision time (in seconds); G^2^, Wilks likelihood ratio chi-square; df, degrees of freedom; BIC, Bayesian information criterion. L, Level factor; B, block-type factor,

*** p < 0.001,

** p < 0.01,

* p < 0.05.

If we consider the rate parameters for Phase1 of response selection, then it is clear that the component rate, μ_*tl*_, for the information at the target level is larger when the target level is global compared to local, *F*_(1, 15)_ = 19.1, *p* < 0.001, η^2^_*p*_ = 0.569. The component rate, μ_*nl*_, for the non-target level differs as well between the levels, *F*_(1, 15)_ = 8.34, *p* < 0.05, η^2^_*p*_ = 0.357. It is also larger for the global target level than for the local one. Here, however, this means that information at the local non-target level has a stronger effect on the global target level than vice versa, and corresponds to the corresponding interactions in the mean data. Taken together, both effects largely outweigh each other, which explains why there was no global advantage. Although information at the global target level produced a larger early activation, information at the local non-target level was more difficult to ignore.

The parameters also demonstrate that the component rates for the non-target level are larger for randomized target levels, *F*_(1, 15)_ = 12.3, *p* < 0.01, η^2^_*p*_ = 0.450, which reflects the fact that the interference between the levels was generally increased when the participants had frequently to switch attention between the levels.

The values of the parameter of late stimulus selection, μ_CLB_, show that the rate was reduced when the target levels were randomized, *F*_(1, 15)_ = 735, *p* < 0.001. This suggests that level uncertainty not only impaired early selection (filtering), but also content-to-level binding. The increased difficulty of binding is also reflected by the confusion errors. A closer look at the details of the simulations revealed that level confusions were practically absent in the constant-level condition (<0.2%), but occurred on about 1% of the trials when target level was randomized. Although the latter rate is still relatively low, it should be noted that task confusions almost always produced errors for incongruent stimuli and mainly occurred for slow responses. In contrast, task confusions had no negative effects for congruent stimuli. Thus, task confusions are one reason why, under randomized levels, accuracy for slow responses to incongruent stimuli did not reach the same high level than that for congruent ones.

The rate for late selection also differed between the target levels, *F*_(1, 15)_ = 48.9, *p* < 0.001. It was higher for the local target level. Moreover, there was a significant two-way interaction between target level and block type, *F*_(1, 15)_ = 22.3, *p* < 0.001, indicating that binding the global target level to its information suffered more from a randomized target level than the binding of the local target level to its information.

Finally, concerning the rate, μ_*RS2*_, for response selection in Phase 2, it was, on average, larger for the global than for the local target level, *F*_(1, 15)_ = 5.27, *p* < 0.05, and reduced under randomized target levels, *F*_(1, 15)_ = 16.8, *p* < 0.001. However, there was also a significant interaction between these two factors, *F*_(1, 15)_ = 7.66, *p* < 0.05, showing that randomizing the target level had a larger negative effect for the global than for the local target level. As a result, under randomized target levels the rate for the global level was even numerically smaller than that for the local one.

Taken together, the modeling results not only nicely reflect the mean data, they also allow one to interpret the results in more detail. It can be seen that information at the global level strongly contributed to response selection in both phases. However, local information intensely interfered with global information, which outweighed the stronger global activation. Moreover, with respect to late selection, the global level suffered more from randomized target levels than the local level. This means, according to the CLB theory, that the level-to-content binding was less efficient for the global level, compared to local, when the target level varied across trials. The process was not only slower, its output rate for response selection was also smaller, compared to the local level.

#### The SSP model

According to the SSP model (White et al., 2011) stimulus selectivity improves gradually with RT. Similar to the DSTP model, it assumes that response selection proceeds by a diffusion process, and that each item provides some perceptual evidence *p* in favor of its associated response. For the present simulations it was assumed that there were only two effective items: the global letter, and the local letter. The weight for each letter is determined by the proportion of attention allocated to the level of that letter. Selectivity, and consequently the drift rate for incongruent stimuli, improves continuously as the relative amount of attention allocated to the target level is gradually increased over time. This is achieved by shrinking the diameter of the attentional “spotlight” at a linear rate, *r*_*d*_, from its initial size, *sd*_0_, to a minimum (0.001). Because the total amount of attention always sums up to 1, performance for congruent stimuli does not change with RT. Altogether, the SSP model has 5 free parameters (see Table 2).

**Table 2:** **Parameter estimates obtained by fitting the SSP model to the distributional data of the four conditions in the experiment**.

	**Parameters**							
**Target level**	***p***	***A/B***	***r_d_***	**s*_d0_***	**t*_er_***	***G*^2^**	***df***	***BIC***
**CONST.**								
Global	0.2754	0.0546	0.0319	1.340	0.3191	34.3 (32.8)	13 (11)	64.8 (75.5)
Local	0.2654	0.0549	0.0316	1.354	0.3182	27.5 (26.1)	13 (11)	58.0 (68.8)
**RAND.**								
Global	0.2512	0.0605	0.0135	1.143	0.3124	41.5 (27.9)	13 (11)	72.0 (70.5)
Local	0.2165	0.0574	0.0298	1.345	0.3229	24.0 (22.3)	13 (11)	54.5 (64.9)
	B^*^		B^*^; L^*^; B×L^*^					

p, perceptual input; A/B, response thresholds; r_d_, rate of decrease in spotlight; s_d0_, spotlight width; t_er_, mean non-decision time (in seconds); G^2^, Wilks likelihood ratio chi-square; df, degrees of freedom; BIC, Bayesian information criterion. The numbers in parenthesis represent the fit performance for the alternative SSPc model (see text for details). L, Level factor; B, block-type factor,

* p < 0.05.

For the present objective, the important characteristic of the model is that the rate for response selection increases gradually with time, at least for incongruent stimuli, due to an improving selectivity of attention. Attention might thereby operate on stimulus location, spatial frequencies, or even both.

#### Fit results for the SSP model

**Figure 6:**
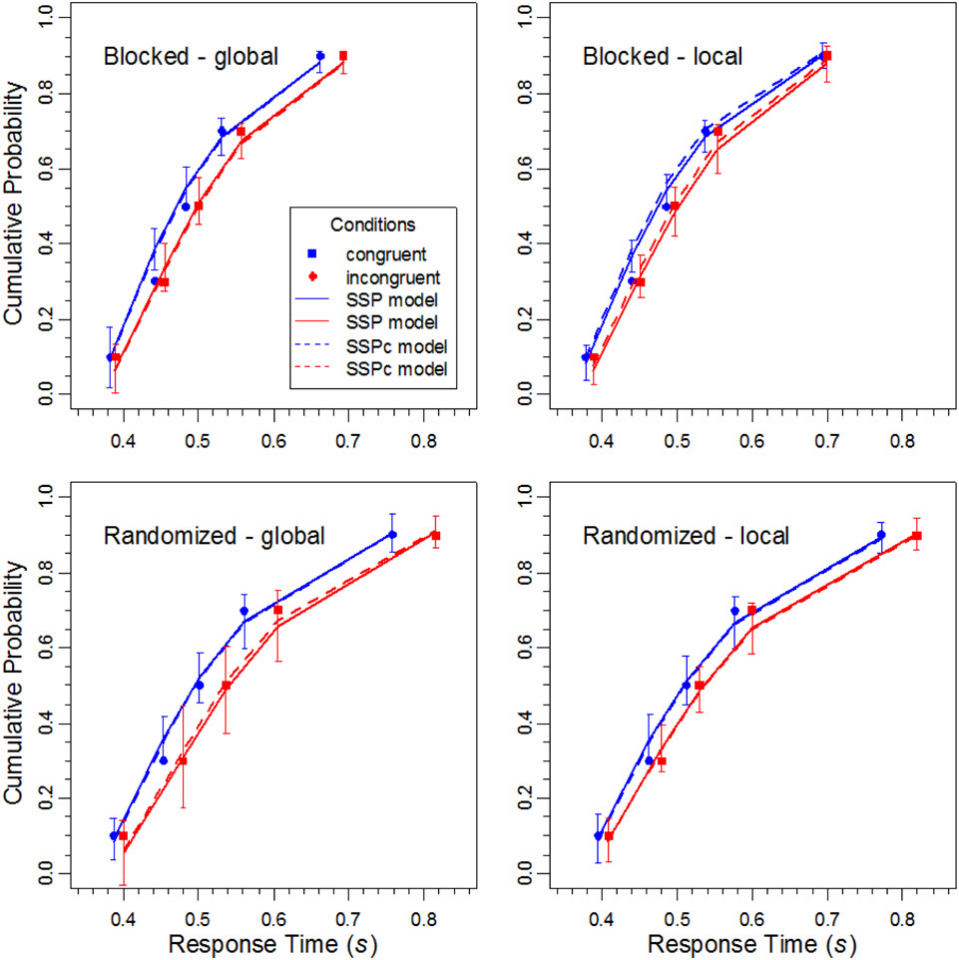
**The data points show the cumulative distribution functions for the RTs of correct responses for the different conditions in the experiment, and solid lines represent the corresponding performance of the SSP model**. The error bars represent the 95%-confidence intervals of the theoretical functions, estimated from the results of the jackknife procedure. The dashed lines show the performance of the SSPc model (see Text for details).

**Figure 7:**
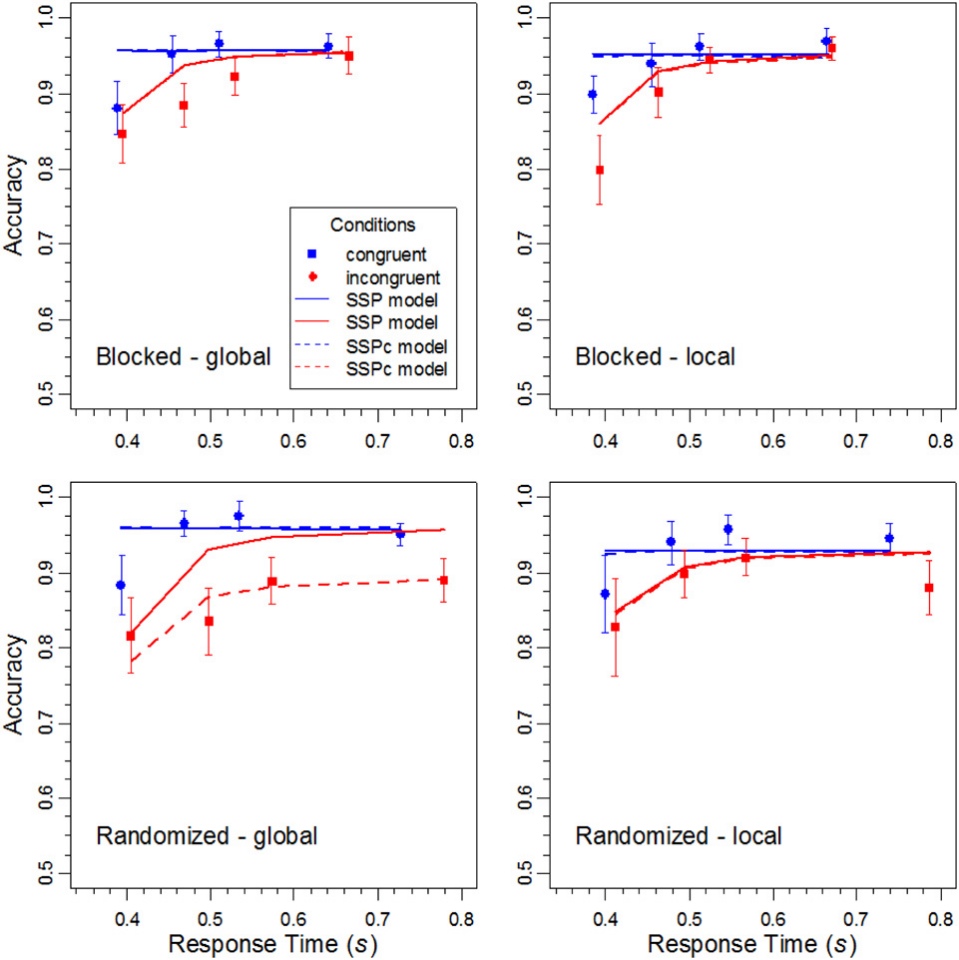
**The data points represent the conditional accuracy functions for the different conditions in the experiment, and the error bars show the respective 95%-confidence intervals**. The solid and dashed lines represent the performance of the SSP model and SSPc model, respectively.

The SSP model was fit to the distributional data with the same procedure as the DSTP model. The obtained mean parameters and the corresponding goodness-of-fit measures are given in Table 2. Obviously, the fit of the SSP is generally worse (in terms of *G*^2^) than that for the DSTP model. In Figures 6 and 7 the obtained theoretical CDFs and CAFs are shown as solid line graphs, respectively. As can be seen by inspecting the CAFs, the SSP model has some difficulties to adapt to the different slopes in the different conditions. It seems that the gradual-improvement mechanism is not very flexible in this respect. Tables 1 and 2 also provide the BIC (Bayesian information criterion) model-selection statistics (Schwarz, 1978), which takes the number of model parameters into account. According to this statistics, the model with the smaller BIC should be preferred. As can be seen, because the SSP has fewer free parameters than the DSTP model, its BIC is slightly superior for one condition (local target level under randomized levels).

The statistical analysis of the parameters revealed (see Table 2) that the perceptual evidence provided by the letter items was significantly lower under randomized than under constant target levels, *F*_(1, 15)_ = 4.64, *p* < 0.05, η^2^_*p*_ = 0.236. Moreover, the rate, *r_d_*, at which the spotlight shrunk was reliably smaller in the randomized condition, *F*_(1, 15)_ = 7.71, *p* < 0.05, η^2^_*p*_ = 0.339, and for the global relative to the local level, *F*_(1,15)_ = 5.33, *p* < 0.05, η^2^_*p*_ = 0.262. However, there was also a significant interaction between block type and target level, *F*_(1,15)_ = 4.64, *p* < 0.05, η^2^_*p*_ = 0.236, indicating that the shrinking rate was especially small for the global level under randomized target levels. Inspection of Figure 7 reveals that for this condition the SSP model produced a relatively low accuracy for fast responses to incongruent stimuli. For slow responses, however, the model predicts the same high accuracy than for congruent stimuli, which substantially differs from the data and leads to the relatively poor fit for this condition.

That accuracy for incongruent stimuli converges to that for congruent ones is an inherent feature of the SSP model. Accordingly, it cannot account for non-converging CAFs. In contrast, as we have seen, the DSTP model has no problems in this respect. This is due, among others, to the possibility of content-to-level binding errors that can occur at the late stage of stimulus selection. The late binding mechanism nicely corresponds to the idea of an abruptly changing selectivity. However, one of the reviewers suggested that non-converging CAFs might also be explained by a gradual-improvement account if one assumes goal (target level) confusions. For the present case, one could assume that the goal was forgotten on some trials during the cue-stimulus interval, and that, therefore, the non-target level was processed, which produced errors also for slow responses. That performance was reduced mainly for the global target level can be explained by additionally assuming that shrinking the spotlight to the local level was easier, which motivated the participants to select it by default.

To see how the SSP model performs if such a goal-confusion mechanism is included, I extended the model accordingly. First of all, an additional parameter was implemented that represents the probability of goal confusion. Furthermore, because the shrinking rate differed significantly between the target levels, two instead of one such parameters were needed. That is, I used one parameter, *r_dt_*, for representing the shrinking rate in case the target level is processed, and another parameter, *r_dn_*, if the non-target level was erroneously chosen. Thus, the extended model, which I refer to as “SSPc” model, has 7 parameters.

Fitting the SSPc model to the averaged data revealed a goal-confusion rate of 9.9% for the global condition under randomized levels. The shrinking rates for the global and local level in this condition were 0.0117 and 0.0312, respectively. For the other three conditions the confusion rate was practically zero. The dashed lines in Figures 6 and 7 show the corresponding theoretical functions. Obviously, the fit to the CAF for the incongruent global condition under randomized levels improved substantially. Accuracy remained relatively low even for slow responses and did not approach that for responses to congruent stimuli.

In Table 2 the performance measures for the SSPc model are represented by the values in parenthesis. As can be seen, the goodness-of-fit (*G*^2^) improved substantially for the critical third conditions, but improved only slightly for the other conditions. Nevertheless, performance remained generally worse than that of the DSTP model. Moreover, due to the additional two parameters, the BIC values are now generally larger for the SSPc model. Thus, the improvement in fit by assuming confusions did not outweigh the overall costs for the additional parameters.

## General discussion

The present study was concerned with the question of how relevant information is selected from hierarchical objects. Based on previous results, it was hypothesized that attentional selectivity should improve during response selection. Consequently, accuracy for incongruent stimuli should increase with RT. To examine whether this prediction holds, a global/local experiment was conducted, in which the difficulty of information selection was additionally varied by blocking vs. randomizing the target level. The obtained CAFs (conditional accuracy functions) clearly show that accuracy for incongruent stimuli was low after stimulus onset, but then increased with RT (see Figure 4). This indicates that attentional selectivity improved during response selection.

According to the DSTP model (Hübner et al., 2010) the increase in selectivity results from two selection stages: an early filtering stage with low selectivity and a late stage with high selectivity. For the present task it was further assumed that late selection proceeds by content-to-level binding. In any case, stimulus selectivity improves discretely in the DSTP model. However, an increasing accuracy with RT could also be due to a gradually improving selectivity, as assumed by the SSP model (White et al., 2011). Therefore, to investigate which account is more appropriate, the two models were fit to the data. Comparing the performance measures of the two models (see Tables 1 and 2) revealed that the goodness-of-fit (*G*^2^) of the SSP model was generally worse than that of the DSTP model. However, because the SSP model has fewer parameters, the model-selection criterion BIC was slightly smaller in one condition (local target level under randomized levels). Nevertheless, taken all conditions together, the DSTP model accounts better for the global/local data than the SSP model.

The DSTP model outperformed the SSP model especially in the global condition under randomized target levels. On corresponding incongruent trials accuracy did not approach that for congruent ones, even for the slowest responses. According to the DSTP model, one reason for this result are level confusions. The reduced efficiency of late selection under a variable target level caused an increased number of level confusion (binding errors). That is, on some trials the letter at the non-target level was selected for response selection in Phase 2, which almost always produced a slow error. That such binding errors indeed occur in global/local tasks has been demonstrated in various studies by applying a masking procedure (e.g., Hübner and Volberg, 2005; Flevaris et al., 2010; Kruse and Hübner, 2012). The fact that the CAFs for the local target level converged in the present case, despite randomization, can be explained by assuming that level-to-content binding was easier for the local level, which largely prevented binding errors. This assumptions is also supported by the variation of other parameters.

Whereas the possibility of late selection errors is a basic property of the DSTP model, the SSP does not have such a mechanism. Therefore, it generally predicts the same high accuracy for slow responses to congruent and incongruent stimuli, which, obviously, was empirically not the case. However, it was possible to implement a similar mechanism by assuming that the target level (goal) can completely be forgotten on some trials and that the easier level is then selected by default. With this extension the fit improved substantially, but only for the critical condition. For the other conditions the possibility of level confusions had practically no effect. As a consequence, the overall improvement of fit performance did not outweigh the costs (in terms of BIC) for the additionally needed parameters. Thus, even after including a mechanism for level confusion in the SSP model, the DSTP model remains the superior model.

The fit of the DSTP model to the data provides a detailed picture of the contribution of the assumed processes. By considering the component rates for local and global information, it is possible to assess the contributions of the levels to response selection in its first phase, i.e., before late stimulus selection took place. The size of these parameters reflect the efficiency of sensory filtering or target-level biasing at the early stage of information selection. As can be seen in Table 1, the component rates for the target levels were substantially larger than those for the non-target levels. Nevertheless, there was also a difference between the levels. If the target level was global, then the corresponding rate was larger than when it was local, which indicates that more attention was allocated to the former than to the latter level. However, at the same time, the component rate for the local non-target level was larger than that for the global non-target level. This means that irrelevant local information was more difficult to filter out or to ignore than irrelevant global information.

Randomizing the target levels affected both early and late stimulus selection. Filtering out irrelevant information was generally more difficult under this condition. That late selection was more difficult when the target level changed frequently is reflected by the decreased rates for the late stimulus-selection process as well as by a reduced output rate of that stage, i.e., by the decreased rates for response selection in Phase 2. The negative effect was especially strong for the global target level.

Taken together, the present data clearly show that stimulus selectivity improves during response selection in global/local tasks. Concerning the question of how this improvement develops in time, it was found that the DSTP model, which assumes a discrete improvement, produced a better fit than the SSP model, which exemplifies a gradual improvement. The discrete selection mechanism of the DSTP model and the corresponding possibility of binding errors accounted especially well for the decrease in accuracy under randomized target levels. However, this feature does differentiate qualitatively between the two improvement accounts. After extending the SSP model by a mechanism that produces goal (target level) confusions, it also predicted a similar reduction in accuracy, although its overall fit remained poorer than that of the DSTP model. Interestingly, the goal-confusion mechanism also comprises a discrete selection step, but at an earlier stage than assumed by the DSTP model. Thus, the question of whether level confusions occur before or during response selection or both offers a further way to investigate how stimulus selectivity improves. Up to know, assuming a discrete improvement of attentional selectivity accounts best for the performance in the global/local task, at least quantitatively and for the present study. Future research will show whether this predominance can be generalized to other phenomena in global/local processing such as level-repetition effects, hemispheric asymmetries, etc. (Hübner and Volberg, 2005).

### Conflict of interest statement

The author declares that the research was conducted in the absence of any commercial or financial relationships that could be construed as a potential conflict of interest.
